# Impact of Short-Cut SWCF Yarn on Conductivity and Electrical Heatability of Silicone–MWCNT Composites

**DOI:** 10.3390/ma14247841

**Published:** 2021-12-18

**Authors:** Kristin Trommer, Minoj Gnanaseelan

**Affiliations:** FILK Freiberg Institute gGmbH, Meißner Ring 1-5, 09599 Freiberg, Germany; minoj.gnanaseelan@filkfreiberg.de

**Keywords:** MWCNT composites, SWCNT yarn, silicone, Joule heating, conductive polymer composites, spread coating, electrical heating

## Abstract

The incorporation of MWCNTs in polymer systems up to the percolation range renders them electrically conductive. However, this conductivity is not high enough for heating applications in the low-voltage range (<24 V). The combination of nanoscaled MWCNTs with microscaled short SWCNT fibers that was investigated in this study causes an abrupt rise in the conductivity of the material by more than an order of magnitude. Silicone was used as a flexible and high-temperature-resistant matrix polymer. Conductive silicone coatings and films with SWCF contents of 1.5% to 5% and constant MWCNT contents of 3% and 5% were developed, and their electrical and thermal properties in the voltage range between 6 and 48 V were investigated. The electrical conductivity of 3% MWCNT composite materials rose with a 5% addition of SWCFs. Because of this spike in conductivity, output power of 1260 W/m^2^ was achieved, for example, for a 100 µm thick composite containing 3% MWCNT and 4% SWCF at 24 V with a line spacing of 20 cm. Thermal measurements show a temperature increase of 69 K under these conditions. These findings support the use of such conductive silicone composites for high-performance coatings and films for challenging and high-quality applications.

## 1. Introduction

In recent years, there has been a paradigm shift toward functional polymer-based composites owing to their unique properties. Their flexibility, stretchability, light weight, impact resistance, fatigue resistance, easy processability, affordability, etc., make polymer composites unique compared to their inorganic counterparts. To add to these assets, current polymer composites attain functionalities, such as energy harvesting, energy storage [1], sensing [2], actuating [3], anti-bacterial [4], anti-fouling [5], self-cleaning [6], photo-catalysis [7], shape memory [8], self-healing [9], electrical heating [10], etc., through tailored approaches. The electrical heatability of polymer composites is of special interest because this is of use in several fields, such as the medical, automotive, electronics, aerospace, sports and leisure, and clothing industries. Conventional electrical heating elements are based on copper or stainless steel [11]. These materials are strong and have high electrical conductivity, thereby providing good performance. However, these pros are accompanied by practical cons. For example, they make elements very brittle and they suffer a considerable loss of efficiency when even a single heating element is accidentally cut. Nanotechnology helps to overcome these deficits by incorporating nanoparticles in a polymer matrix, wherein the nanoparticles act as nanoheaters and generate heat all over the composite [12]. Even if such heating elements are damaged by pin holes, slight cuts, or cracks, the performance of the nanoheater composite is not compromised because the electrons flow around these discontinuities. To develop such composites, electrically conductive nanoparticles, such as carbon black, silver nanowire, graphene, graphite, carbon nanotube (CNT), etc., are required. Composites should exhibit high electrical conductivity and low specific heat capacity to be suitable for heating applications [13]. Carbon nanotube composites are ideal candidates owing to their high aspect ratio, as they can easily form percolated networks at low contents. Multiwall carbon nanotubes (MWCNTs) are the cost-efficient choice among CNTs. However, there is an inherent limitation to the conductivity of MWCNTs. Therefore, if some MWCNTs could be replaced by another high-conductive filler, then a cost-efficient, high-performance composite can be produced.

In this study, conductive silicone composites were prepared using a hybrid filler system as a coating, as well as freestanding films. The electrical properties of such coatings and films were investigated. Subsequently, their heating behavior and the output power were analyzed.

## 2. Materials and Methods

### 2.1. Chemicals

Non-substituted multiwalled carbon nanotubes (MWCNTs) available in large quantities in the market were used as electrically conductive nanoparticles (Nanocyl^TM^ NC 7000, Sambreville, Belgium). They appear as a black powder of particle agglomerates on a millimeter scale. The primary particles are 9 nm in diameter and 10 µm in length. MWCNTs were used in combination with short-cut yarn composed of single-walled carbon nanotubes (SWCFs) by Conyar B. V. (sc-Conyar™, Arnhem, Netherlands). The yarn (611 dtex) was made of 18 filaments, the length of each section of yarn was 3 mm, and the resistivity was approximately 0.43 µΩm.

Both carbon additives were used without further modification.

To achieve a good mix of the carbon additives with the polymer matrix, polymer solutions were preferred. Silicone was dissolved in an organic solvent (toluene) and then conductive additives were mixed into it using a three-roll mill to obtain a paste-like conductive dispersion. This paste was then further diluted until the viscosity was suitable for the knife-coating process.

An addition-crosslinking 2 K liquid silicone was chosen, in which the vinyl component (Elastosil 6250 F, Wacker Chemie AG, Munich, Germany) can be vulcanized with 1–3% crosslinker (W, Wacker Chemie AG; Si–H component, Munich, Germany). The hardness is reported as 36 Shore A, the tensile strength as 5.0 N/mm^2^, and the elongation at a break as 350% in the data sheet. Vulcanization usually occurs for 2–4 min at temperatures above 150 °C.

### 2.2. Preparation of Dispersions Containing MWCNT Particles and SWCF

The primary CNT particles possess a diameter in the nanoscale range and a high aspect ratio, and they are present as particle agglomerates. The breaking up of the existing MWCNT agglomerates and the exfoliation of the MWCNT bundles, which are prerequisites for network formation, cause a sharp increase in viscosity. A directed shear mixing process in a three-roll mill, which is preceded by a homogenization of the agglomerates with a stirrer, is suitable for the optimal dispersion and distribution of the MWCNT particles in a silicone matrix. As MWCNTs and SWCFs are highly flexible and have thread-like structures, they were processed together with a 120-EH-250 three-roll mill from Exakt Advanced Technologies GmbH (Norderstedt, Germany). The three-roll mill has two separately adjustable roll nips. Likewise, the speed of the three rolls can be adjusted individually, when the ratio of the speeds v1:v2:v3 is set at 1:3:9. In principle, the three-roll mill can be operated in gap or force mode. Either the nip can be precisely defined and the force required to press the material through the nip varied, or the force with which the rolls are pressed against each other can also be defined, while the nip width is adjusted automatically during the passage of the material through the nip. When setting the parameters for the roll speed and the nip, it is important to achieve a directed, moderate shearing. By this means, the following objectives were attained during the mixing process:

- Breakdown of the MWCNT agglomerates without damaging the primary particles;
- Disentanglement of the MWCNT clusters or exfoliation of the MWCNT bundles;
- Formation of an MWCNT network in the polymer matrix.

The optimal settings for the processing of MWCNT-containing silicone composites in our previous work served as the initial parameters [14,15,16]. Only slight changes were necessary to obtain a homogeneous particle dispersion. Both additives, MWCNTs and SWCFs, were incorporated into the silicone matrix simultaneously. Typically, 100 g of liquid silicone was placed in a container. One after the other, the MWCNTs and SWCFs were added in portions and premixed. Subsequently, the compound paste was prepared on the three-roll mill in four passages, with the nip widths as stated in Table 1. The resulting paste was diluted with toluene to a viscosity suitable for coating and a 1% crosslinker (related to silicone) was added. The paste was again homogenized on a three-roll mill with a nip setting of 60 µm:20 µm and then degassed at −0.8 bar for 30 min to remove entrapped gasses that might have created bubbles in the resulting film.

### 2.3. Preparation of Conductive Silicone Composite Films

The conductive films were prepared using a lab coater (Zürich, Switzerland) from Mathis AG following the delamination process. In this method, the silicone hybrid compound is coated onto a release paper using a knife-over-roll arrangement, and then dried and vulcanized. After solidification, the conductive silicone films can be delaminated from the transfer paper, yielding a self-standing film. For all samples, a constant thickness of 100 µm was chosen to ensure the properties of the films were comparable. Because of the limitation of the lab coating device, the maximum sample size that can be produced by this method is 25 cm × 25 cm.

In a typical procedure for manufacturing 100 µm thick films, a plain TPX-coated release paper ( Schöller, Weißenborn, Germany) is used. Two layers of coating are applied, and each layer is dried at 70 °C for 2 min and vulcanized together at 180 °C for 6 min. The hybrid silicone composites are characterized in the form of coatings on the paper and free-standing films.

### 2.4. Measurement of Electrical Properties

#### 2.4.1. Resistivity, Conductivity

According to ISO 2878, the surface resistance was determined using a LORESTA GX device (Fa. N & H-Technologie, Willich, Germany) with a 4-PIN measuring head (MCP-T700, Willich, Germany). The resistivity was calculated by taking into account the sample thickness, the dimensions of the sample, and the measuring position. Conductivity results from the inverse of the resistivity. The samples were prepared by punching out 10 cm × 10 cm sections of film and conditioning them at 23 °C in 27% relative humidity for 24 h. The final value was calculated from the mean value of three measurements, each made in longitudinal and transverse directions.

#### 2.4.2. Electrical Power per Unit Area

**Figure 1:**
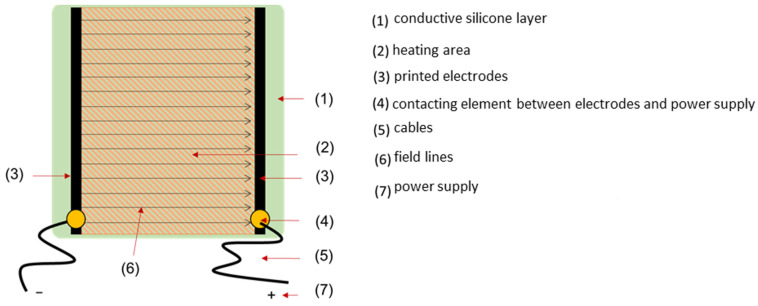
Setup of the contacted silicone hybrid material.

The prerequisite for heating the samples is to electrically contact their surfaces, for which two electrodes in the shape of a 3 mm wide line were printed on the surfaces of the coatings or films at variable distances parallel to each other. A highly conductive silver compound with a Ag content >85% and a silicone-based binder was used. This pasty mixture was suitable for application by screen printing. Voltage was applied to these printed electrodes and the current flowing between them was measured to yield the *I–U* graphs. The current flow leads to the heating of the sample because of the Joule effect. The sample setup is shown schematically in Figure 1. The calculation of the electrical power per unit area was carried out using Equation (1).

(1) $$P=\frac{U*I}{A}$$

*U*–Voltage [V], *I*–current flow [A] and *A*–sample area [m^2^]. 

#### 2.4.3. Heating Behavior

A VarioCAM hr research thermographic system (InfraTec GmbH, Dresden, Germany), of which the thermal resolution at 30 °C is at least 0.06 K, was used to investigate the heatability. The test setup consisted of a thermally insulating base covered with a Teflon glass fabric and a black textile on which the sample was placed. The measurement was carried out in an enclosure made of thermo-Styrofoam as protection against air movement, with a measuring field of 15 cm × 15 cm centered on the sample (20 cm × 20 cm). The mean value of the temperature in the measuring field was used for the comparative evaluation of the maximum heating temperatures of different samples. For the temperature distribution on the sample, the image recordings of the thermal-imaging camera were used. The reference measurement, which is necessary for determining the temperature difference, was taken outside of the heatable area. The temperature of the reference point was almost constant over the measurement period (at 23 °C, SD <0.5 °C).

#### 2.4.4. Micromorphological Characterization

Cross-sections of the composites were prepared with a sharp razor blade. In case of imaging of the SWCF powder, the powder was just placed over the double sided conductive tape. The cross-section of the composites and powders were then coated in a thin layer (3 nm) of gold and observed using a Quanta 250 FEG scanning electron microscope (FEI, Dresden, Germany). A secondary electron detector (SE2) was used for capturing images at an operating voltage of 10 kV with a working distance of approximately 10 mm.

## 3. Results and Discussion

### 3.1. Electrical Properties of Hybrid Composites

**Figure 2:**
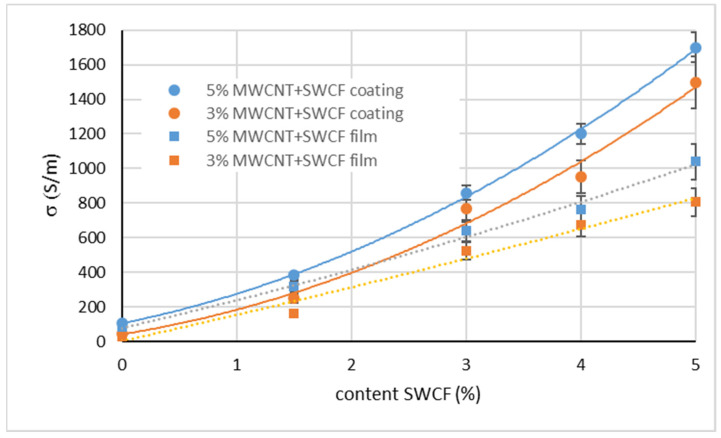
Conductivity of silicone films containing 5% (blue) and 3% (orange) MWCNT and different contents of SWCFs, as measured on the transfer paper.

The MWCNT content of the hybrid silicone films was kept constant at 5% and 3% and the effect of SWCFs was systematically investigated in the range of 1.5–5%. The conductivity determined for the films on the transfer paper (coating) is shown in Figure 2 (dots and solid lines). The conductivity for 3% and 5% MWCNT particle content without any SWCF, caused by the percolation of the MWCNT in the silicone matrix, was 46 S/m and 115 S/m, respectively. The addition of 1.5% SWCF already caused a significant increase in conductivity from approximately 115 S/m (without SWCFs) to 370 S/m (5% MWCNT), or from 46 S/m to 240 S/m (3% MWCNT). With the addition of 5% SWCF, maximum values of conductivity of approximately 1700 S/m (5% MWCNT) and approximately 1500 S/m (3% MWCNT) were obtained. The conductivity of most of these composites is better than that of other CNTs, carbon black (CB), and graphene-based elastomer composites. The existing literature about the electrical conductivity of elastomer/MWCNT composites reports significantly lower conductivity at similar loadings, which is shown in Table 2. For instance, TPU/5 wt % MWCNT composites have a conductivity of just 9.5 S/m, which is an order of magnitude less than that of the corresponding composite in this work [17]. This can be attributed to the excellent mixing quality achieved using a three-roll mill, rather than a twin-screw extrusion process. There are also other elastomeric composites based on MWCNTs that show very low conductivity compared to the composite in this work (Table 2). IR/4.8 wt % MWCNT and NR/5.6 wt %, which were produced using a two-roll mill mixing step, exhibit conductivity that is 3–4 orders of magnitude lower. Carbon nanostructures (CNSs), which have a branched structure of CNTs, could provide better conductivity for TPU at 4 wt % loading of 40 S/m. The use of a hybrid filler system (MWCNT/cellulose nanocrystals (CNCs)) also did not yield high conductivity. Lin et al. implemented a modified approach for using ionic liquid (IL) to disperse and align the MWCNTs and they achieved good conductivity of 1000 S/m. In our work, it was possible to obtain much higher conductivity of 1700 S/m using SWCFs. This large synergistic effect can be explained, on the one hand, by the higher electrical conductivity of SWCFs compared to the network-forming multiwalled MWCNTs. On the other hand, the short cuts of the SWCF-based yarn can be effectively dispersed because of their good wettability and flexibility. This applies to both the separation of the individual fibers and their homogeneous distribution in the composite, which obviously result in intensive cross-linking of the conductive MWCNT particles. Although the milling step can cause an orientation of the high-aspect particles, the electrical measurements show isotropic behavior. The following processing, including mixing and homogenization, leads to isotropic distributed particles. After delaminating the coatings from the transfer paper, the films showed less conductivity. The dotted curves in Figure 2 illustrate the course of conductivity. This effect occurred for films containing 3% MWCNT and 5% MWCNT in the same intensity. The higher the SWCF content, the higher the decrease in conductivity. Delaminating the coatings from the transfer paper results in a change in the shape of the films. Volume shrinkage occurs because of the transition from a liquid to a dry solid state during vulcanization. In the state of a coating, the silicone hybrid layer is fixed on the substrate (transfer paper). After delamination, the elastic properties of the silicone cause a shrinkage in the length and width of the now-separated layers by approximately 3%. We think that this change in dimensions provokes changes in the alignment of the various conductive high-aspect-ratio particles and results in a less intensive interaction. Consequently, the conductivity decreases. Nevertheless, the conductivity is still very high compared to that of films without a synergist and they are suitable for heating purposes.

### 3.2. Heating Behavior of Hybrid Composites

**Figure 3:**
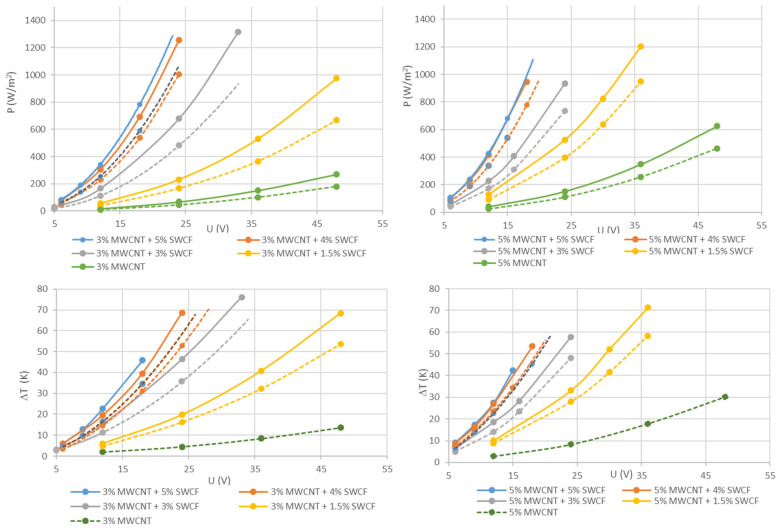
**Top:** Electrical area output of silicone hybrid layers containing 3% (**left**) or 5% (**right**) MWCNT and variable SWCF content depending on the voltage (conducting distance 20 cm). **Bottom**: Temperature change as a function of the voltage (solid line: values for coatings; dashed line: values for films)**.** To avoid confusion, if the error was less than 10%, it is not shown in the plot.

To investigate the electrical area output power and the heatability of silicone hybrid layers with different contents of MWCNTs and SWCFs, the samples were contacted with electrodes that were 3 mm in width and 20 cm apart. The thickness of the conductive layer was in the range of 95 to 110 µm because of the limited accuracy of the manufacturing procedure. As the carbon nanoparticles form the electrically conductive network, their content is of great importance for obtaining high electrical conductivity. Figure 3 shows the results of the electrical area output power (top) and the achievable temperature difference (bottom) for hybrid films containing a low (3%) and a high (5%) MWCNT content with 1.5%, 3%, 4%, and 5% synergists (SWCF). Both properties were measured at voltages in the range of 6–48 V. The curves show that both the temperature and the area output power increase many times when the SWCF content is increased in a matrix of equal MWCNT content. Because of the different conductivity, the coatings that remain on the paper show higher values for the electrical area output power and temperature increases compared to the free-standing films. In the two graphs describing the electrical area output power (Figure 3, top), the reference without SWCF is marked in green. For example, at a voltage of 24 V, the coating with 3% MWCNT shows an area output power of 44.5 W/m^2^, whereas the coating that contains an additional 1.5% SWCF shows 230 W/m^2^, with 3% 680 W/m^2^, and with 4% SWCF 1260 W/m^2^. A further increase in the SWCF content does not result in significantly higher area output power (orange and blue curves). The results for coatings containing 5% MWCNT show the same tendency, but the absolute values for the area output power are much higher. To ensure safe handling, films and coatings with the high additive content of 5% MWCNT and ≥4% SWCF were tested up to 24 V (4% SWCF) and 16 V (5% SWCF), respectively. The measuring device that allows a current up to 2200 mA sets this limitation. Therefore, the value for 5% MWCNT and 4% SWCF in the coating at 24 V outside the measuring range was extrapolated, resulting in 1650 W/m^2^. Because of the high electrical output, both the coatings and films are suitable for heating applications. The graphs of the temperature differences correlated with the voltages show the maximum temperature difference that can be obtained (Figure 3, bottom). As expected, the highest performance is achieved with a coating that contains 5% MWCNT and 4% SWCF. It heats up by 28 K when applying a low voltage of 12 V, which is appropriate for close to the body applications. A comparable temperature difference of about 30 K can be achieved at different voltages using different formulations. For example, 16 V is required for composites with 5% MWCNT and 3% SWCF, or 23 V for composites with 5% MWCNT and 1.5% SWCF. As is obvious from the curves in the diagrams, the achievable temperature differences of 4% and 5% SWCF are very close to one another for hybrid materials with 3% MWCNT. If the hybrid materials contain 5% MWCNT, there is no measurable difference between those with an additional content of 4% or 5% SWCF. We conclude that an optimal synergistic effect is achieved with 4% SWCF.

**Figure 4:**
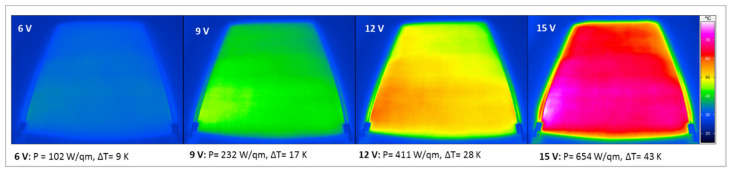
Thermographic images of the silicone films containing 5% MWCNT and 5% SWCF at voltages between 6 and 15 V.

The heating of the materials was captured visually using an IR camera. The images show homogeneous heating of the surface. All films and coatings heat up in the low-voltage range below 24 V. For example, Figure 4 shows the IR images of the silicone hybrid film with 5% MWCNT and 5% SWCF at different voltages. With a low voltage of 15 V, a temperature change of 43 K is achieved.

### 3.3. Micromorphological Investigations

**Figure 5:**
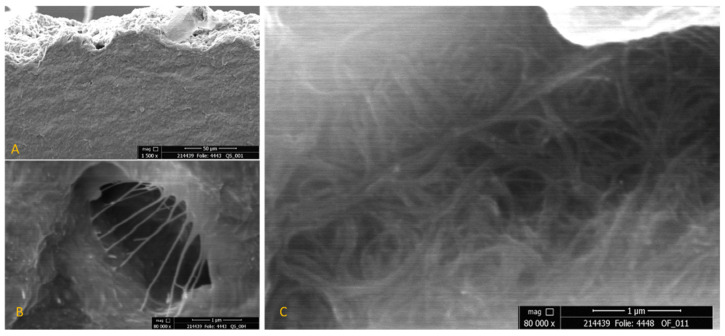
SEM images of embedded CNT particles; (**A**) cross-section in a low magnitude; (**B**) alignment of polymer-covered CNT bundles in a microbubble; and (**C**) CNT alignment in the polymer matrix.

**Figure 6:**
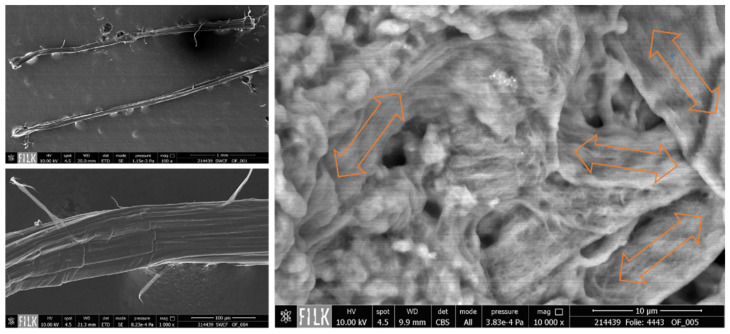
SEM images of a single SWCF (**left**), and SWCFs embedded in the PU matrix (**right**).

To examine the morphological structure of the composites in more detail, we captured SEM images of the cross-section and the surface. Because of the extreme difference in the sizes of nano- and microscaled particles, CNTs and SWCFs cannot be observed in a single image. Figure 5A shows an overview of the cross-section in a low magnification of 1500×, which depicts the formation of homogeneous film. The sample contains 3% CNT and 5% SWCF. Because the carbon particles are wrapped with silicone, the CNT bundles are more visible in places with defects such as micropores or cracks (Figure 5B). This embedment of carbon particles in the matrix also results in low contrast; because of this, images must be recorded with a high magnification of 80,000× (Figure 5C). Such a high magnification helps to capture the random arrangement of the CNTs. This accords with the electrical properties, where an isotropic behavior was perceived. Figure 6 shows the single SWCF (left) and the SWCF embedded in the polymer (right). The arrows indicate the isotropic alignment of the short-cut fibers. There is no preferred orientation. At a relatively low magnification of 10,000×, only a SWCF wrapped in polyurethane is visible and the CNT bundles are not visible.

## 4. Conclusions

In this work, the possibility of enhancing the extrinsic electrical conductivity of MWCNT-containing silicone coatings and films using a highly conductive flexible SWCF was investigated. The incorporation of MWCNT and SWCF particles in silicone solutions using a three-roll mill enables a homogeneous distribution of the particles and their alignment in an electrically conductive network. The electrical conductivity in the silicone matrix of approximately 115 S/m (5% MWCNT) achieved by this method can be increased by more than an order of magnitude with the addition of 1.5–5% SWCF as a synergist. A conductivity of 1700 S/m was attained for such a hybrid material with 5% MWCNT and 5% SWCF. After applying parallel silver-based polymeric electrodes to the surface, voltages between 6 and 48 V were applied, which generated an electric field area that heats up to a certain temperature. With a given film thickness of 100 µm and with electrodes at a distance of 20 cm, it is possible to establish the required temperature differences by varying the MWCNT or SWCF contents, as well as the voltage. For example, a composite containing 3% MWCNT and 4% SWCF at 24 V heats up by 69 K. Using a composite with 3% MWCNT and 1.5% SWCF at 48 V, or with 5% MWCNT and 1.5% SWCF at 35 V, the same temperature increase of 69 K can be achieved. Therefore, it is possible to adapt the system to specific application requirements. The low-voltage applications (<24 V) offer great potential, especially in the mobile lightweight sector, where functional and lightweight materials are particularly advantageous.

## Figures and Tables

**Table 1:** Processing parameters of the three-roll mill.

Passage	Nip 1 Width (µm)	Nip 2 Width (µm)	Roll Speed (U/min)
1	150	50	200–300
2	40	20	200–300
3	15	10	200–300
4	5	5	200–300

**Table 2:** Electrical conductivity of elastomer composites.

Composite	Electrical Conductivity (S/m)	Reference
IR/CB (14 wt %)	0.02	[18]
NR/CB (13 wt %)	0.001	[19]
SBR/NR/graphene (8 vol %)	0.2	[20]
NR/8 vol % graphene	2	[21]
SBR/NR/3.2 vol % graphene	3	[20]
IR/CNT (4.8 wt %)	0.01	[18]
NR/CNT (5.6 wt %)	0.001	[19]
SBR/BR/CNT (2 wt %)/SiO_2_ (28 wt %)	0.15	[22]
TPU/5% CNT	9.5	[17]
TPU/20% CNT/IL	1000	[23]
TPU/4 wt % CNS	40	[24]
NR/CNT-CNC (4 vol %)	1	[25]
Silicone (5% CNT/5% SWCF)	1700	this work

## Data Availability

The data presented in this study are available on request from the corresponding author.

## References

[1] Nair S.S., Mishra S.K., Kumar D. (2021). Review–polymeric materials for energy harvesting and storage applications. Polym.-Plast. Technol. Mater..

[2] Adhikari B., Majumdar S. (2004). Polymers in sensor applications. Prog. Polym. Sci..

[3] Hu L., Zhang Q., Li X., Serpe M.J. (2019). Stimuli-responsive polymers for sensing and actuation. Mater. Horiz..

[4] Luo H., Yin X.Q., Tan P.F., Gu Z.P., Liu Z.M., Tan L. (2021). Polymeric antibacterial materials: Design, platforms and applications. J. Mater. Chem. B.

[5] He Z., Lan X., Hu Q., Li H., Li L., Mao J. (2021). Antifouling strategies based on super-phobic polymer materials. Prog. Org. Coat..

[6] Kumar A. (2021). Smart bioinspired anti-wetted surfaces: Perspectives, fabrication, stability and applications. Curr. Res. Green Sustain. Chem..

[7] Cantarella M., Impellizzeri G., Di Mauro A., Privitera V., Carroccio S.C. (2021). Innovative Polymeric Hybrid Nanocomposites for Application in Photocatalysis. Polymers.

[8] Xia Y., He Y., Zhang F., Liu Y., Leng J. (2021). A review of shape memory polymers and composites: Mechanisms, materials, and applications. Adv. Mater..

[9] Del Prado-Audelo M.L., Caballero-Florán I.H., Mendoza-Muñoz N., Giraldo-Gomez D., Sharifi-Rad J., Patra J.K., González-Torres M., Florán B., Cortes H., Leyva-Gómez G. (2021). Current progress of self-healing polymers for medical applications in tissue engineering. Iran. Polym. J..

[10] Chen L., Zhang J. (2021). Designs of conductive polymer composites with exceptional reproducibility of positive temperature coefficient effect: A review. J. Appl. Polym. Sci..

[11] Kim H., Kim H.S., Lee S. (2020). Thermal insulation property of graphene/polymer coated textile based multi-layer fabric heating element with aramid fabric. Sci. Rep..

[12] Park J. (2020). Functional fibers, composites and textiles utilizing photothermal and joule heating. Polymers.

[13] Liu L., Peng S., Niu X., Wen W. (2006). Microheaters fabricated from a conducting composite. Appl. Phys. Lett..

[14] Trommer K., Morgenstern B., Petzold C. (2015). Preparing of Heatable, CNT-Functionalized Polymer Membranes for Application in Textile Composites. Mater. Sci. Forum.

[15] Heise M., Trommer K., Gnanaseelan M., Wela-Wallenschus I., Mahlkow A. (2020). Electrically Conductive and Flexible Polymer Films as Substrates and Power Transmitter for Wearable Electronics. Curr. Trends Polym. Sci..

[16] Gnanaseelan M., Trommer K., Gude M., Stanik R., Przybyszewski B., Kozera R., Boczkowska A. (2021). Effect of Strain on Heating Characteristics of Silicone/CNT Composites. Materials.

[17] Christ J.F., Aliheidari N., Ameli A., Pötschke P. (2017). 3D printed highly elastic strain sensors of multiwalled carbon nanotube/thermoplastic polyurethane nanocomposites. Mater. Des..

[18] Chen J., Li H., Yu Q., Hu Y., Cui X., Zhu Y., Jiang W. (2018). Strain sensing behaviors of stretchable conductive polymer composites loaded with different dimensional conductive fillers. Compos. Sci. Technol..

[19] Natarajan T.S., Eshwaran S.B., Stöckelhuber K.W., Wießner S., Pötschke P., Heinrich G., Das A. (2017). Strong Strain Sensing Performance of Natural Rubber Nanocomposites. ACS Appl. Mater. Interfaces.

[20] Lin Y., Liu S., Chen S., Wei Y., Dong X., Liu L. (2016). A highly stretchable and sensitive strain sensor based on graphene–elastomer composites with a novel double-interconnected network. J. Mater. Chem. C.

[21] Zhan Y., Lavorgna M., Buonocore G., Xia H. (2012). Enhancing electrical conductivity of rubber composites by constructing interconnected network of self-assembled graphene with latex mixing. J. Mater. Chem..

[22] McNally T., Pötschke P. (2011). Polymer-Carbon Nanotube Composites: Preparation, Properties and Applications.

[23] Lin L., Liu S., Fu S., Zhang S., Deng H., Fu Q. (2013). Fabrication of Highly Stretchable Conductors via Morphological Control of Carbon Nanotube Network. Small.

[24] Sang Z., Ke K., Manas-Zloczower I. (2018). Interface Design Strategy for the Fabrication of Highly Stretchable Strain Sensors. ACS Appl. Mater. Interfaces.

[25] Wang S., Zhang X., Wu X., Lu C. (2016). Tailoring percolating conductive networks of natural rubber composites for flexible strain sensors via a cellulose nanocrystal templated assembly. Soft Matter.

